# Association of health literacy with quality of life and health outcomes among school-age children in Japan: A cross-sectional study

**DOI:** 10.1371/journal.pone.0324456

**Published:** 2025-05-20

**Authors:** Mika Ninohei, Hiroki Sugimori, Naoko Ito, Ataru Igarashi, Mika Kigawa, Junko Miyazawa, Maki Hirao, Keiko Suzuki, Takeshi Odajima, Takeo Nakayama

**Affiliations:** 1 Faculty of Sports and Health Science, Department of Nursing, Daito Bunka University, Higashimatsuyama city, Saitama, Japan; 2 Department of Health Data Science, Yokohama City University, Yokohama City, Kanagawa, Japan; 3 Graduate School of Health and Social Service, Kanagawa University of Human Services, Yokosuka City, Kanagawa, Japan; 4 Faculty of Nursing, Department of Nursing, Josai International University, Togane City, Chiba, Japan; 5 Faculty of Sports and Health Science, Department of Health Science, Daito Bunka University, Higashimatsuyama, Saitama, Japan; 6 Japanese Red Cross-kanto-koshinetsu Block Blood Center, Tokyo, Japan; 7 Department of Health Informatics, School of Public Health, Kyoto University, Kyoto City, Kyoto, Japan; Kyushu University, JAPAN

## Abstract

Health literacy is a modifiable determinant of health with the potential to enhance public health. An association between health literacy and health-related quality of life has been reported. Although each country has developed their own original health literacy scales, the assessment of adolescent health literacy using the Health Literacy Scale for School-Aged Children has not yet been studied in Japan. In this study, we aimed to clarify the factors associated with adolescents’ health literacy and examine the relationship between health literacy, health-related behaviors, and health-related quality of life in Japan. Participants were recruited by a research company using registered monitors (1st- to 3rd-year junior high school students and their mothers living in Japan in August 2023). Multivariate regression analysis was performed using the total EuroQoL Five Dimensions, Youth Version scores. SAS software was used for data analysis. Overall, 1,854 adolescents and their mothers participated in the online survey. Factors associated with Health Literacy Scale for School-Aged Children included physical activity, sleep conditions in health-related behaviors, parental communication, parental health literacy, and health-related quality of life. Furthermore, parental health literacy was associated to children’s quality of life. Our study showed the influence of family variables, highlighting the need for tailored approaches that consider parents’ health literacy levels.

## Introduction

Health literacy (HL) is defined as “the personal, cognitive, and social skills which determine the ability of individuals to gain access to, understand, and use information to promote and maintain good health [1] and improve quality of life (QoL) during the life course” [2]. It is categorized into three groups: functional, interactive, and critical HL [1]. Functional HL refers to basic-level skills, involving reading, writing, numeracy, and the ability to understand and use basic information regarding health and medicine [1,3]. Interactive HL describes more advanced social and cognitive skills and refers to the ability to acquire health information through communication and apply it to changing environments [1,3]. Critical HL is the most advanced skill and refers to the ability to critically analyze health and medical information and use it independently. It includes the ability to improve health status through social or political action [1,3,4]. Groups with high HL tend to avoid smoking and drinking alcohol [5], have appropriate exercise habits [5–7], and contribute to preventing the severity of heart disease and diabetes [6–7]. Conversely, groups with low HL tend to practice poor preventive care, exhibit low medication adherence [8], and self-care [9]. A relationship between HL and health behaviors in adolescents has been shown [10–11]. Adolescents with low HL are associated with health problems such as smoking and obesity, and it has been noted that they have particular challenges with communication and critical thinking [10]. HL develops gradually through education and experience from childhood and adolescence [12]. Adolescence is a critical developmental stage marked by numerous physical, emotional, and cognitive changes [13], during which individuals practice and develop essential skills for lifelong well-being [14]. Adolescent HL is influenced by family demographic factors, including parental HL levels, socio-economic status [15], and overall health status. Studies have shown that low parental HL affects children’s health, particularly in areas, such as oral health [16], physical activity [17], nutrition [18].

HL for School-Aged Children (HL-SAC) is a specific scale designed to assess the HL of school-aged children [19]. Studies utilizing HL-SAC have been conducted in 10 European countries, revealing that the majority of adolescents had moderate or higher HL. However, low HL prevalence was reported in Czechia (17.7%), Austria (16.6%), and Germany (16.0%). Additionally, a positive association was found between HL and both the family affluence and self-rated health [20]. An Italian study further highlighted that school satisfaction and teacher support were strongly associated with HL, and parental support and communication were also significant factors [21].

Health-related quality of life (HRQoL) is an index that evaluates the impact of health on daily and social life [22]. HL plays an intermediary role between health knowledge and health behavior [23] and a mediating role in the relationship between HL and HRQoL [24]. HL influences health-related decision-making and has long-term implications for future health and quality of life. Although previous studies have examined the relationship between HL and HRQoL, no studies have yet applied the HL-SAC scale in Japan.

Therefore, in this study, we aimed to clarify the factors related to adolescents’ HL and the relationship between HL, health-related behaviors, and HRQoL.

## Materials and methods

### Study design

In this cross-sectional study, a web-based questionnaire was used to collect data. The study results are reported according to the Strengthening the Reporting of Observational Studies in Epidemiology guidelines [25].

### Sampling and participants

Participants were recruited using registered monitors of a research company and stratified sampling by sex, age, and region of 1st- to 3rd-year junior high school students and their mothers living in Japan. The sample size was calculated using G*Power version 3.1.9.6 (Mac OS X 10.7 to 14, Heinrich Heine Universität, Düsseldorf, Germany). With an effect size *f* ^2^ of 0.25, α error probability of 0.05, and a power (1-β error probability) of 0.8, the estimated number of participants was approximately 1,800, accounting for the possibility of missing responses owing to inappropriate answers. Participants received an e-mail containing the URL of the survey. The informed consent document included all required information posted on the first page of the online questionnaire, and those who consented to the study could proceed to the questionnaire. An online survey was conducted from August 21–22, 2023. Written informed consent was obtained from both children and their mothers (opt-out procedure). This study was approved by the Daito Bunka University Research Ethics Committee (approval number: DHR23–007).

### Eligibility criteria

The study included children aged 12–15 years (junior high school students, 1st to 3rd grade) and their mothers who consented and could participate with their children. Those who did not provide consent to participate in the study or lacked Internet access were excluded.

### Measurements

#### HL for parents and children.

HL was measured using the HL-SAC [19] and the Japanese version of the 14-item Health Literacy Scale (HLS-14) [26]. After obtaining permission from the original author, the Japanese version of the HL-SAC was translated. Subsequently, a pilot study involving 114 adolescents (aged 12‒15 years) was conducted to validate the scale (GFI, 0.804; comparative fit index, 0.727; root mean square error of approximation, 0.181) and reliability (theoretical knowledge: Cronbach’s α, 0.75; practical knowledge: α, 0.44; critical thinking: α, 0.73; self-awareness: α, 0.70; citizenship: α, 0.52; and HA-SAC: total α, 0.83). The HL-SAC is an HL scale for children that consists of 10 items, with two items representing each of the five components: (1) theoretical knowledge, (2) practical knowledge, (3) critical thinking, (4) self-awareness, and (5) citizenship. The items are scored on a Likert scale ranging from 1 (not at all true) to 4 (absolutely true). The total score is categorized into three levels: low (10–25), moderate (26–35), and high (36–40).

Parental HL was measured using the Japanese version of the HLS-14, developed by Ishikawa et al. [23]. This scale consists of three components: (1) functional, (2) communicative, and (3) critical HL. The items were scored on a Likert scale ranging from 1 (not at all true) to 5 (exactly true), with higher scores indicating higher HL.

#### EuroQoL five dimensions, youth version for children.

The EuroQoL Five Dimensions, Youth Version (EQ-5D-Y) is a specific instrument for school-aged children (8‒15 years). A Japanese version of the scale was developed, and the reliability and validity have been confirmed [27,28]. The EQ-5D-Y instrument comprises five dimensions: 1) mobility; 2) looking after oneself; 3) performing usual activities; 4) experiencing pain or discomfort; and 5) feeling worried, sad, or unhappy. The EQ-5D-Y descriptive system comprises five dimensions: “walking about” (“mobility”), “looking after oneself” (“self-care”), “performing usual activities” (“usual activities”), “experiencing pain or discomfort” (“pain or discomfort”), and “feeling worried, sad, or unhappy” (“anxiety or depression”). Each dimension comprises three levels: no problems, some problems, and a lot of problems. The items were scored on a Likert scale ranging from 1 (no problem) to 3 (a lot), with higher scores indicating better HRQoL.

#### Demographic factors, parental economic condition, and education level.

Demographic factors included the age, sex, and region of both the children and mothers. Parental economic condition was assessed based on household income, classified into three levels: low (<4 million JPY), medium (4–8 million JPY), and high (>8 million JPY), according to the Comprehensive Survey of Living Conditions [29]. Education level was categorized into four levels: junior and senior high school, professional school and junior college, university, and postgraduate education.

#### Health status for children.

Body mass index (BMI) was classified by WHO classification for Japan: underweight, < 18.5 kg/m^2^; normal weight, 18.5–24.9 kg/m^2^; and overweight, ≥ 25.0 kg/m^2^. Information regarding current illnesses was obtained and recorded as the presence or absence of illnesses under treatment.

#### Health-related behaviors for children.

In this study, we assessed health-related behaviors in children, including physical activity (frequency of exercise and sports other than school classes, categorized as more than once a week or less than once a week), sleep duration time (>6 or <6 hours), self-reported restfulness (sufficient or insufficient), and the frequency of skipping breakfast (>4 or <4 times a week). Since the survey period was during the summer vacation, the children were instructed to answer the questionnaire regarding their lifestyle habits during the last month before the summer vacation. The questionnaires were developed based on data from the National Health and Nutrition Survey [30].

#### Parental communication for children.

The survey to assess communication patterns and investigate their relationship with family dynamics was developed based on the National Health and Nutrition Survey [29]. The primary focus was to examine the frequency of eating meals with family (>4 or <4 times a week) and whether the child engages in conversation with family members during meals (yes or no).

### Statistical analysis

Based on the median total score, HL-14 scores were divided into two levels (high and low), while HL-SAC was divided into three levels (high, moderate, and low). Continuous variables with a non-normal distribution were analyzed using the Mann–Whitney U test. Categorical variables were analyzed using the chi-squared test. Responses to the EQ-5D-Y were converted into index values using the Japanese value set [27]. Multiple linear regression analysis was performed using the total EQ-5D-Y score as the dependent variable. To assess multicollinearity, the correlation coefficient and variance inflation factor between each independent variable were confirmed. The independent variables included socioeconomic factors, the presence of disease, HL-SAC, and HLS-14. SAS software Version 9.4 (SAS Institute, Cary, NC, USA) was used for data analysis.

## Results

Children (1,854 children) and their mothers participated in the online survey. No missing values were reported, and all data were anonymized by a research agency before collection and analysis. The demographic characteristics of the participants are presented in Table 1. The ages of the children ranged from 12 to 15 years (1st to 3rd grade), and the mean age of their mothers was 44.2 years. Half of the mothers had an education level below university. Household income distribution in this survey was as follows: 34.4% of households had an income of <6 JPY million, 19.0% had an income of 6–8 JPY million, and 19.0% had an income of ≥8 JPY million. Compared to national data (median income: JPY 7.31 million) [26], 41.5% of the respondents reported an income above the median (Table 1).

**Table 1:** Demographic Characteristics.

Variables	Total (n = 1,854)
Sociodemographic factors Children’s Age	
12‒13 years (1st grade)	618 (33.3)
13‒14 years (2nd grade)	618 (33.3)
14‒15 years (3rd grade)	618 (33.3)
Children’s sex	
Male	927 (50%)
Female	927 (50%)
Region	
Hokkaido	80 (4.3)
Tohoku	94 (5.1)
Kanto	611 (33.0)
Chubu	385 (20.8)
Kinki	359 (19.4)
Chugoku	94 (5.1)
Shikoku	51 (2.8)
Kyushu	180 (9.7)
Household income (JPY 1 million)	
< 2	33 (1.8)
2– < 4	187 (10.1)
4– < 6	418 (22.5)
6– < 8	352 (19.0)
8– < 10	199 (10.7)
10– < 15	132 (7.1)
15– < 20	18 (1.0)
≥ 20	13 (0.7)
Others	189 (10.2)
Mother’s age (Mean, SD)	44.2 (4.4)
Parental HL (Mean, SD)	52.7 (7.9)
Mother’s education level	
Junior and senior high school	503 (27.1)
Professional school and junior college	694 (37.4)
University and post-graduation	646 (34.9)
Others	11 (0.6)
Health status for children	
BMI [kg/m^2^]	
Underweight (BMI < 18.5 kg/m^2^)	602 (32.5)
Normal weight (BMI 18.5–24.9 kg/m^2^)	1,166 (62.9)
Overweight (BMI > 25.0 kg/m^2^)	86 (4.6)
History of present illness	
Yes	322 (17.4)
Dental	119 (37.0)
Mental health	46 (14.3)
Asthma	37 (11.5)
Anemia	30 (9.3)
Hypertension	23 (7.1)
Others	67 (20.8)
No	1,532 (82.6)

BMI, body mass index; JPY, Japanese Yen; SD, standard deviation; HL, Health literacy

Table 2 presents the mean HL-SAC scores according to sex. HL-SAC was scored by two items from each of the five components. The mean score for female individuals was higher than that for male individuals in all dimensions. The total mean (standard deviation [SD]) score was 26.28 (5.0) points. The highest total mean score was for practical knowledge (6.0), while theoretical knowledge (4.94) and critical thinking (4.95) had low scores. No significant differences were found between the sexes.

**Table 2:** Mean HL-SAC Scores by Sex.

HL-SAC dimensions	Males (n = 927)	Females (n = 927)	Total (n = 1854)	*P*-value ^*1*^
	Mean (SD)	Mean (SD)	Mean (SD)	
Theoretical knowledge	4.90 (1.2)	4.98 (1.1)	4.94 (1.2)	0.166
I am confident that… 1)I have good information about health	2.24 (0.7)	2.29 (0.7)	2.26 (0.7)	
5)When necessary, I am able to give ideas on how to improve health in my immediate surroundings	2.66 (0.7)	2.69 (0.6)	2.67 (0.6)	
Practical knowledge	5.95 (1.1)	6.04 (1.0)	6.0 (1.1)	0.106
4) I can follow the instructions given to me by healthcare personnel (e.g., nurse, doctor)	3.14 (0.6)	3.1 (0.6)	3.15 (0.6)	
7) When necessary, I find health-related information that is easy for me to understand	2.8 (0.6)	2.8 (0.6)	2.84 (0.6)	
Critical thinking	4.92 (1.2)	4.98 (1.2)	4.95 (1.2)	0.332
3) I can compare health-related information from different sources	2.41 (0.7)	2.45 (0.7)	2.43 (0.7)	
9) I can usually figure out if some health-related information is right or wrong	2.50 (0.7)	2.52 (0.7)	2.51 (0.7)	
Self-awareness	5.30 (1.2)	5.32 (1.2)	5.32 (1.2)	0.776
8) I can judge how my behavior affects my health	2.77 (0.6)	2.75 (0.6)	2.76 (0.6)	
10) I can give reasons for choices I make regarding my health	2.53 (0.7)	2.56 (0.7)	2.54 (0.7)	
Citizenship	5.03 (1.2)	5.09 (1.2)	5.06 (1.2)	0.294
1)When necessary, I am able to give ideas on how to improve health in my immediate surroundings (e.g., a nearby place or area, family, friends)	2.38 (0.7)	2.44 (0.7)	2.41 (0.7)	
6)I can judge how my own actions affect the surroundings natural environment	2.64 (0.7)	2.64 (0.6)	2.64 (0.6)	
Total score	26.12 (5.2)	26.42 (4.7)	26.28 (5.0)	0.206

^1^student’s t-test, HL-SAC; health literacy scale for school-aged children; SD, standard deviation.

Table 3 presents the three levels of HL-SAC. The total HL-SAC score was categorized as follows: low (10‒25 points), moderate (26‒35 points), and high (36‒40 points). The majority of children had moderate HL (96.7%), and only 0.5% had low HL.

**Table 3:** Level of HL-SAC by Sex.

	N (%)	Low HL	Moderate HL	High HL
Total	1,854 (100)	9 (0.5)	1,794 (96.7)	51 (2.8)
Male	927 (50)	7 (0.8)	892 (96.2)	28 (3.0)
Female	927 (50)	2 (0.2)	902 (97.3)	23 (2.5)

HL, health literacy; HL-SAC, health literacy scale for school-aged children.

Table 4 presents the results of the multiple linear regression analysis. Significant factors included mother’s education level (university and postgraduatation; estimate: 0.408, *P* < 0.0001); physical activity (never per week; estimate: -1.422, *P* < 0.0001); sleeping condition (insufficient; estimate: -1.545, *P* < 0.0001); frequency of eating meals with family (<4 times a week; estimate: -1.167, *P* = 0.029); conversation with family while eating (no; estimate: -1.663, *P* = 0.0004); and HLS-14 score (estimate: 0.127, *P* < 0.0001).

**Table 4:** Relationships between the HL-SAC index values and age, sex, health status, health-related behavior, mother’s education level, household income, and HLS-14.

	Estimate	Standard error	*P-*value^*1*^
Intercept Sociodemographic factors			
Children’s age			
12‒13 years (1st grade)	–	–	–
13‒14 years (2nd grade)	–	–	–
14‒15 years (3rd grade)	0.011	0.132	0.929
Children’s sex			
Male	–	–	–
Female	0.314	0.250	0.209
Mother’s education level			
Junior and senior high school	–	–	–
Professional school and junior college	–	–	–
University and post-graduation	0.408	0.099	<.0001
Household income (JPY million)			
< 4			–
4– < 8			
≥ 8	0.235	0.145	0.106
Health status for children Any illness currently being treated			
Yes	–	–	–
No	0.026	0.325	0.934
BMI			
Underweight (BMI < 18.5 kg/m^2^)			
Normal weight (BMI 18.5–24.9 kg/m^2^)			
Overweight (BMI > 25.0 kg/m^2^)	0.290	0.229	0.205
Health-related behavior Physical activity			
≥ once in a week			
Never	-1.422	0.260	<.0001
Sleeping time			
≥ 6 h			
< 6 h	0.112	0.376	0.764
Sleep condition			
Sufficient			
Insufficient	-1.545	0.336	<.0001
Skipping meal			
< 4 times a week			
≥ 4 times a week	-0.288	0.432	0.504
Parental communication			
Frequency of eating meal with family			
≥ 4 times a week			
< 4 times a week	-1.167	0.534	0.029
Conversation with family while eating			
Yes			
No	-1.663	0.465	0.0004
Parental HL (HLS-14)	0.127	0.015	<0.0001

^1^Multiple linear regression analysis.

HL-SAC; Health Literacy Scale for School-Aged Children; BMI, body mass index; JPY, Japanese Yen; HLS-14, 14-item Health Literacy Scale.

Table 5 summarizes the percentage of reported problems in the EQ-5D-Y. The percentage of children who responded “no problem” to the dimensions related to mental health (“experiencing pain or discomfort” [68.9%] and “feeling worried, sad, or unhappy” [59.8%]) was lower than that for other dimensions (“doing usual activities” [89.1%], “mobility” [71.3%], and “looking after oneself” [93.9%]).

**Table 5:** Distribution of Responses of the EQ-5D-Y.

Response n (%)			
EQ-5D-Y dimensions	A lot	Some	No
Mobility	89 (4.8)	443 (23.9)	1,322 (71.3)
Looking after myself	16 (0.9)	97 (5.2)	1,741 (93.9)
Doing usual activities	38 (2.0)	164 (8.9)	1,652 (89.1)
Having pain or discomfort	58 (3.1)	519 (28.0)	1,277 (68.9)
Feeling worried, sad, or unhappy	66 (3.6)	679 (36.6)	1,109 (59.8)

EQ-5D-Y, EuroQoL five dimensions Youth Version.

Table 6 presents the results of the multiple linear regression analysis. Significant differences in children’s EQ-5D-Y scores were observed based on sex (female; 0.015, *P* = 0.049), the absence of any illness currently being treated (no; estimate: -0.061, *P* < 0.0001), HL-SAC (estimate: -0.005, *P* < 0.0001) and HLS-14 scores (estimate: -0.001, *P* < 0.0007). Lower EQ-5D-Y scores indicate better HRQoL. These results suggest a positive and significant association between child HL and parental HL.

**Table 6:** Relationships between children’s EQ-5D-Y index values and age, sex, health status, mother’s education level, household income, HL-SAC, and HLS-14.

	Estimate	Standard error	*P*-value^*1*^
Intercept Sociodemographic factors			
Children’s age			
12‒13 years (1st grade)	–	–	–
13‒14 years (2nd grade)	–	–	–
14‒15 years (3rd grade)	-0.002	0.004	0.620
Children’s sex			
Male	–	–	–
Female	0.015	0.006	0.049
Mother’s education			
Junior and senior high school	–	–	–
Professional school and junior college	–	–	–
University and post-graduation	0.002	0.005	0.581
Household income (JPY million)			
< 4			–
4– < 8			
≥ 8	-0.008	0.004	0.069
Health status for children Any illness currently being treated			
Yes	–	–	–
No	-0.061	0.010	<0.0001
BMI			
Underweight (BMI < 18.5 kg/m^2^)			
Normal weight (BMI 18.5–24.9 kg/m^2^)			
Overweight (BMI > 25.0 kg/m^2^)	-0.007	0.007	0.285
HL-SAC	-0.005	0.0008	<0.0001
Parental HL (HLS-14)	-0.001	0.0005	0.0007

^1^Multiple linear regression analysis.

BMI, body mass index; HL-SAC; Health Literacy Scale for School-Aged Children; HLS-14, 14-item Health Literacy Scale; EQ-5D-Y, EuroQoL five dimensions, Youth Version; JPY, Japanese Yen.

## Discussion

### Association between adolescent’s HL and health-related behavior

The mean total HLS-14 score obtained in this study was similar to that from a national survey of individuals aged 40–49 years using the same scale [26]. The mean HL-SAC score (SD) for the total sample was 26.2 (5.0) points, with female individuals having a higher mean HL-SAC score than male individuals.

In a report from seven European countries [31], the HL-SAC scores of 25,425 adolescents aged 13–15 years indicated that the percentage of low HL ranged from 9.0% to 18.4%, and the percentage of high HL ranged from 13.1% to 35.0%. Additionally, a report from 10 European countries, including 14,590 adolescents aged 15 years, showed that 13.3% had low HL, 67.2% had moderate HL, and 19.5% had high HL. The mean (SD) total score was 30.78 (5.34), with the highest score being 33.93 (4.78) and the lowest being 29.88 (5.32) points [20].

These results suggest that the findings in Japan (low: 0.5%, high: 2.8%) show a lower bias toward low and high HL levels. The scores in Japan were higher than those observed in the European samples. Furthermore, the mean scores for theoretical knowledge and critical thinking in the present study were lower than those reported in European countries, indicating that critical thinking and the application of health information are particularly low in Japan. Results from the European Consortium Health Literacy Questionnaire, which assessed 1,054 Japanese adults aged 20–69 years, suggested that the skills required to “evaluate” and “apply” health information are challenging to acquire [32].

In this study, parental factors (education level and HL level), health-related behaviors (physical activity, sleep conditions), and parental communication (frequency of family meals, conversation during meals) were found to be associated with HL-SAC. Previous studies have also reported that sociodemographic characteristics and social class factors such as sex, race, family affluence, parental education levels, and economic status are linked to children’s HL and health outcomes [15,20,21,33]. HL is based on lifestyle habits during early childhood, and studies have been reported that examine the association between parental HL levels and children’s health behaviors, with a particular focus on food and nutrition, oral hygiene, and medical conditions [34]. Additionally, the quality of communication with parents and family support plays a crucial role in children’s health. Previous studies have shown that the quality of parental communication and family support are significant predictors of HL-SAC [21]. In this study, the frequency of family meals and communication with parents were found to be associated with HL. Previous studies have highlighted the importance of the school and community environment, including factors such as school satisfaction, teacher support, neighborhood livability, and available facilities [21,24]. These factors are relevant to child health and should be considered in future research.

### Association of HL-SAC and -health-related HRQoL

The percentage of children who responded “no problem” to dimensions related to mental health was low across the five dimensions. Conversely, more than 80‒90% reported “no problem” for “looking after oneself” and “doing usual activities.” A Japanese EQ-5D-Y report involving 3,636 children and adolescents aged 8‒15 years [28], a multinational study including 2,809 children and adolescents aged 8‒19 years from European and African countries [22], and a study of 1,229 adolescents aged 12–18 years from Spain [35] all showed low scores for the dimension related to mental health. Moreover, Japanese children had lower average mental health scores than did adolescents. In adults, age, sex, and chronic diseases tend to affect EQ-5D-Y scores [36], but in children and adolescents, there is no difference in scores by sex or age, and it has been shown that the scores are influenced by some childhood diseases and household income [28]. This study did not show an association with household income, but did show an association with parental HL levels. Many studies have reported an association between socioeconomic status and HL levels, and parental attributes have been reported to affect children and adolescent’s HL [20–21]. Although no direct association was found in this study, such background may have had an influence. Additionally, insufficient sleep time and poor sleeping conditions were linked to lower mental health scores. A previous study showed that sleeplessness negatively affected both HRQoL and mental health [37]. Increasing parental HL is expected to foster healthy lifestyle habits in children, ultimately improving HRQoL.

### Limitations

This study has some limitations. The EQ-5D-Y was used because the research participants were a relatively healthy general population without severe disabilities or illnesses. However, previous studies have shown that its sensitivity is low, it cannot assess psychosocial health, the three-level option has weak detection power for moderate health disorders, and it lacks discrimination. Additionally, the ceiling effect is high, with scores tending to cluster at the upper end. This study included participants registered with a survey company and may have represented a population with high e-HL, which may have caused selection bias. The findings of cross-sectional studies using the HL-SAC among middle school students are not available for Japan; therefore, further research should be conducted to compare the results across populations and countries.

## Conclusions

The purpose of this study was to clarify the factors related to adolescents’ HL and examine the relationship between HL, health-related behaviors, and HRQoL. Factors associated with HL-SAC included physical activity, sleeping conditions, parental communication, parental HL, and HRQoL. Additionally, parental HL was associated with children’s HRQoL, highlighting the influence of family variables. These findings suggest that interventions should be tailored to parents’ HL levels to support adolescent health.

Moreover, insufficient sleep was linked to the EQ-5D-Y dimension related to mental health, underscoring adolescent mental health as a significant concern. The school and community environment, including school satisfaction, teacher support, neighborhood livability, and available facilities, also play an important role and should be considered in future research.
