# Correction: Association of health literacy with quality of life and health outcomes among school-age children in Japan: A cross-sectional study

**DOI:** 10.1371/journal.pone.0349244

**Published:** 2026-05-13

**Authors:** Mika Ninohei, Hiroki Sugimori, Naoko Ito, Ataru Igarashi, Mika Kigawa, Junko Miyazawa, Maki Hirao, Keiko Suzuki, Takeshi Odajima, Takeo Nakayama

There is an error in the second sentence of the third paragraph of the “Association between adolescent’s HL and health-related behavior” subsection of this article’s [1] Discussion section. The correct sentence is: The scores in Japan were lower than those observed in the European samples.

The authors apologize for the error.
